# Addressing vaccination gaps among healthcare workers in sub-Saharan Africa: the role of mandatory Hepatitis B vaccination

**DOI:** 10.1186/s41182-024-00652-x

**Published:** 2024-11-06

**Authors:** Faithful Miebaka Daniel, Bonaventure Michael Ukoaka, Victoria Ezinne Emeruwa, Rosette Chidera Oti-Ashong, Gabriel Oluwafemi Falaiye

**Affiliations:** 1 Community and Clinical Research Division, First On-Call Initiative, No. 4 Freedom Square, Kharkiv, Ukraine; 2 Community and Clinical Research Division, First On-Call Initiative, Nigeria, No. 4-8 Harley Street, Old GRA, Port Harcourt, Nigeria; 3 Medical Services, International SOS. Intels Camp KM12 Aba Expressway, Port Harcourt, Nigeria

**Keywords:** Mandatory vaccinations, Hepatitis B virus, Needlestick injuries, Developing countries, Health personnel

## Abstract

Hepatitis B virus (HBV) poses a significant public health threat, particularly in developing countries with high endemicity but poor vaccination among healthcare workers (HCWs). Needlestick injuries increase HCWs' risk, yet only about 42% of HCWs are fully vaccinated compared to 97% in high-income countries. Challenges to vaccine uptake include availability, demanding schedules with frequent unit rotations hindering access, high cost of acquiring shots, and stock shortages resulting in missed opportunities. Mandatory, cost-free HBV vaccinations for HCWs, supported by legislation, international aid, and digital reminders, could ensure self-protection and safety while contributing to the global objective of eradicating HBV by 2030.

Hepatitis B virus (HBV) constitutes a global public health concern, with two billion people infected worldwide, mainly in Africa and Asia [1]. Healthcare workers (HCWs) are at increased risk, with medical students also vulnerable during cadaveric dissections and more prone to needlestick injuries [2, 3]. Vaccination is the most effective strategy to achieve the global goal of eradicating HBV infection as a public health concern by 2030 [1]. Paradoxically, low- and middle-income countries (LMICs) with the highest endemicity have the poorest uptake among HCWs. For instance, only 42% of HCWs in Nigeria are fully vaccinated, compared to 97% in high-income countries like France [1]. Reasons for poor uptake include stock unavailability, lack of reminders, high cost of receiving doses, and the need for a leave of absence [1].

Percutaneous exposure to HBV in non-immune HCWs is frequently reported, requiring post-exposure prophylaxis (PEP) with Hepatitis B immunoglobulin (HBIG), ideally administered within 24 h [3, 4]. Unfortunately, HBIG is often inaccessible as the financial burden often exceeds the average HCW’s monthly salary, or 'undocumented' treatments are given in the absence of established protocols [3]. In March 2024, a doctor in Nigeria sustained a needlestick injury while treating a patient with HBV infection [4]. The HBIG was unavailable in the entire state where the incident occurred and could only be procured from Abuja, the Nation’s capital, which is 278 km away, and arrived after 24 h [4].

In LMICs, vaccinations have a fee but are optional, and HCWs do not actively seek them. Only a few opt for vaccinations when they are available in their health facilities, and even such availability is irregular. This irregularity in vaccine supply chain has culminated in periodic missed opportunities. Proactive vaccination with comprehensive HCW coverage is needed to address this issue. Globally, vaccinations are mostly optional. However, in certain contexts, such as in Italy, the United States, and the United Kingdom, they have been made compulsory to meet vaccination coverage targets [5, 6]. Various ethical arguments for and against compulsory vaccinations have been proposed. However, the advantages outweigh the ethical pitfalls. Patient autonomy has been at the core of these debates [5–7]. In response to the legal dilemma, it has been posited by the US Supreme Court that the protection of public health takes precedence over individual interests within the confines of reasonable limits [5]. One consensus is that for vaccines to be made compulsory, there must be a reliable supply of effective and safe vaccines and the willingness to take them [5]. Both conditions are fulfilled in SSA regarding HBV vaccination.

In SSA, where collectivist societies predominate, mandatory vaccinations might be less of an ethical conundrum and more acceptable compared to Western individualized societies. In African cultures, the collective good is prioritized over the individual good. Against this backdrop, considering the enormous HBV vaccination gap, it is imperative to adopt compulsory vaccinations in SSA countries. Besides, compulsory vaccinations for hepatitis tend to be more accepted than others, with acceptance as high as 85% in several studies [8]. Compulsory hepatitis vaccinations for HCWs have significant advantages. They ensure the protection of HCWs, a high-risk population who are essential but few in the fragile health systems in most SSA countries. Additionally, it is an important consideration for patient safety, further limiting transmission in the general population. Given that the hepatitis burden is highest, and vaccination coverage is poorest in these regions, mandatory vaccination appears to be a viable solution in addition to other strategies.

Based on these considerations, we recommend free, mandatory vaccination for all HCWs, with expenses covered by training institutions or employers through a legislative act. In the interim, this vaccination should be made a requirement for the accreditation of facilities and issuance of practicing licenses by supervisory medical councils and health ministries. The CDC recommends accelerated vaccinations that can be completed within 21 days, followed by a booster in a year; this can aid the immediate deployment of HCWs. The Occupational Health and Safety Act (OSHA) has similar provisions in the United States. Some developed countries enforce mandatory vaccination for medical students [2]. LMICs can adopt mandatory policies for students and interns before the commencement of clinical exposures, shifting from mere recommendations. Furthermore, HCWs can use low-cost digital tools for appointment reminders. The support of international donor agencies, health ministries, and charities is also needed to make these vaccines available. The expertise of supply chain professionals will be invaluable in ensuring effective management of vaccine supplies to abate stockouts. To address ethical concerns, conscientious exemptions can be given upon satisfaction of administrative requirements that must be robust, to avoid being counterintuitive. The policies should also consider social equity, ensuring that those who opt out do not unfairly benefit from herd immunity without the associated risks of vaccinations. HCWs, like other population groups, may have cultural or personal beliefs about vaccinations, which can influence their willingness to take the Hepatitis B vaccine. For instance, religious or traditional beliefs about health and disease prevention could affect vaccine acceptance. The role of health promotion leveraging good information dissemination through the media can greatly remediate vaccine hesitancies.

In conclusion, adopting compulsory HBV vaccination policies for HCWs in LMICs, particularly in sub-Saharan Africa, is essential for protecting this high-risk group and curbing HBV transmission. This strategy, supported by legislation, international aid, and digital tools, can help achieve the global goal of eradicating HBV as a public health concern by 2030.

## Data Availability

Data sharing is not applicable to this article as no datasets were generated or analyzed during the current study.

## Abbreviations

HBV: Hepatitis B virus
HCW: Health care workers
CDC: Centre for Disease and Control and Prevention
WHO: World Health Organization
HBIG: Hepatitis B immunoglobulin
LMIC: Low- and middle-income countries
OSHA: Occupational Safety and Health Act
